# Stimulation of soy seeds using environmentally friendly magnetic and electric fields

**DOI:** 10.1038/s41598-023-45134-y

**Published:** 2023-10-23

**Authors:** Agata Dziwulska-Hunek, Agnieszka Niemczynowicz, Radosław A. Kycia, Arkadiusz Matwijczuk, Krzysztof Kornarzyński, Joanna Stadnik, Mariusz Szymanek

**Affiliations:** 1 Department of Biophysics, University of Life Sciences in Lublin, Akademicka 13, 20-950 Lublin, Poland; 2 Department of Analysis and Differential Equations, University of Warmia and Mazury in Olsztyn, Słoneczna 54, 10-710 Olsztyn, Poland; 3 Faculty of Computer Science and Telecommunications, Cracow University of Technology, 31-155 Kraków, Poland; 4 Department of Mathematics and Statistics, Masaryk Univeristy, Kotlářská 267/2, 611 37 Brno, Czech Republic; 5 Department of Animal Material Technologies, University of Life Sciences in Lublin, Skromna 8, 20-704 Lublin, Poland; 6 Department of Agricultural, Forest and Transport Machinery, University of Life Sciences in Lublin, Głeboka 28, 20-612 Lublin, Poland

**Keywords:** Ecology, Plant sciences

## Abstract

The study analyses the impact of alternating (magnetic induction B = 30 mT for t = 60 s) and constant magnetic fields (B = 130 mT for t = 17 h) and alternating electric fields (electric current E = 5 kV/cm for t = 60 s) on various growth parameters of soy plants: the germination energy and capacity, plants emergence, the fresh mass of seedlings, protein content (Kjeldahl’s method), and photosynthetic parameters (with MINI-PAM 2000 WALTZ Photosynthesis Yield Analyser and a SPAD-502 Chlorophyll Meter). Four cultivars were used: MAVKA, MERLIN, VIOLETTA, and ANUSZKA. Moreover, the advanced Machine Learning processing pipeline was proposed to distinguish the impact of physical factors on photosynthetic parameters. The use of electromagnetic fields had a positive impact on the germination rate in MERLIN seeds. The best results in terms of germination improvement were observed for alternating magnetic field stimulation in all cultivars (p > 0.05). For the VIOLETTA cultivar an increase (p > 0.05) in the emergence and overall number of plants as well as fresh mass was observed after electromagnetic field stimulation. For the MAVKA and MERLIN cultivars, the concentration of proteins in the leaves was noticeably higher in plants grown from seeds stimulated using a constant magnetic field.

## Introduction

Soy is one of the most important arable crops cultivated worldwide^1–3^. It is a leguminous plant whose symbiosis with *Rhizobium* bacteria enables it to bind nitrogen. This capacity makes for an inexpensive method of preserving soil fertility and improving plant yields^4^. Soy seeds are a valuable source of protein (30–40%) and fat (19–20%), as well as minerals and vitamins, owing to which they play an essential part in human and animal nutrition^5–10^. Soy is often used in food products and medicinal foodstuffs in the East^5,11^, e.g. in the form of sprouts, oil, paste^12^, or soy meal^13^ and soy milk^14^.

Soy production is hindered by poor germination rates and low seed vitality. The vitality of soy seeds is short-lived when compared to other plants, and strongly affected by factors such as storage conditions, mechanical damage, and damage suffered during post-harvest processing^15^. Therefore, it is necessary to employ methods facilitating better seed quality^16^.

A key concern related to achieving higher efficiency in soy cultivation is the improvement of the germination rate, e.g., by employing one of the methods of processing seeds before sowing. The methods discussed in the following article include stimulation with alternating and constant magnetic fields and alternating electric fields^17–21^. Due to their environmental friendliness, the techniques could replace chemical treatment in the future. Other physical methods are also found in the literature, among others: laser light, gamma radiation, etc.^22,23^. The priming of seeds affects the physiological and biochemical properties of the material^23^. Seed priming entails a pre-sowing treatment that affects physiological processes taking place within the seeds. The most common methods include: hydropriming^24^, biostimulant priming^25^, antioxidative priming^26^, and irradiation^27^.

Plants are naturally adapted to living in the magnetic field of our planet. The characteristics of the Earth’s magnetic field obviously depend on the geographical latitude, and its intensity can range from 0 to 67 μT^28^. The studies conducted to date on seed stimulation using magnetic fields have demonstrated significant improvement in terms of plant germination, emergence, growth, and yield. The most commonly identified factors influencing the effectiveness of such treatment include magnetic induction, frequency of alternating fields, seed exposure time, and polarity (north, south)^29^ [Krawiec et al. 2018]. Constant and alternating magnetic fields used for seed stimulation have ranged from 3 mT^30^ to 480 mT^31^, with the exposure time from 4 s to 24 h^32,33^. Magnetic field stimulation was one of the broadly studied methods of seed processing analysed in terms of impact on seed germination, plant growth, and biological parameters^34–36^. The technique remains the subject of considerable research scrutiny, mainly because of its non-invasiveness and environmentally friendly character, as well as its effectiveness in stimulating better seed germination and vigor^37^. Research has also been conducted on the effects of an alternating magnetic field with the ions cyclotron resonance frequency (ICR) of Ca^2+^ on wheat seedlings, and the results evidenced a positive influence on the number of sprouts as well as the wet and dry weight of the seedlings^38^. Stimulating seeds with an alternating electric field increased growth capacity in seven-year-old radish seeds by approx. 80%^39^, and the germination capacity of old tomato seeds (Halicz cultivar) by between 46 and 80%^40^ or even 100%^41^.

From a technical perspective, chlorophyll fluorescence is measured using specialist fluorometers. The method can be used in studies on various aspects of photosynthesis in plant production^42,43^. The method of determining leaf greenness using a portable chlorophyll meter (SPAD-502) can be used for fast and repeatable measurements in various plant species^44,45^.

The research problem explored in this study pertained to determining the impact of soy seed priming with the use of alternating and constant magnetic fields as well as an alternating electric field, on germination and plant emergence, photosynthetic parameters, and protein content in the leaves. The studied variables underwent a two-way ANOVA analysis and relevant hypotheses were formulated in terms of whether the germination energy and capacity of the seeds, plant emergence and number of plants after 30 days, photosynthetic efficiency, electron transport, greenness index, and protein content in the leaves were affected by: H_A0_: electromagnetic field stimulation (alternating and constant magnetic field and alternating electric field), H_B0_: soy cultivar (MAVKA, MERLIN, VIOLETTA i ANUSZKA), and H_AB0_: combined influence of the soy cultivar and electromagnetic field stimulation?

Moreover, the rising importance of advanced Machine Learning methods in science provides an excellent opportunity to use them in research to discover non-trivial relationships in the data. The paper contains a well-thought clustering pipeline to check physical factors’ influence on soy plants.

## Materials and methods

### Plants material

The research material comprised seeds of four soy (*Gylcine max* L.) cultivars: MAVKA, MERLIN, VIOLETA, and ANUSZKA obtained from a 2017 harvest at the Department of Plant Production Technology and Product Science of the University of Life Sciences in Lublin and stored under room conditions. MAVKA is a semi-early cultivar originating from Poland. It tends to ripen evenly and shows resistance to shedding and lodging. MERLIN is a semi-early cultivar originating from Austria, characterised by a high yield potential and high plant protein content. VIOLETTA (VIOLA) is a semi-late cultivar from Lithuania. It produces stable, high yields and is characterised by up to 40% general protein content. It is highly resistant to disease, particularly brown spot and bacterial blight. ANUSZKA (Annuhka) is a very early cultivar from Ukraine. It is characterised by a very short vegetation period of 90–100 days and very high yield potential. In 2018, an experiment was conducted at the Department of Biophysics of the University of Life Sciences in Lublin, Poland (51^*o*^14^′^37^′^N, 22^*o*^32^′^26^′^E).

It entailed two stages: (1) energy and capacity germination of seeds on Petri dishes, and emergence, (2) number of plants, fresh mass, photosynthetic parameters (greenness index (SPAD), photosynthetic efficiency (Y (II)), electron transport rate (ETR)) and protein content in pot-grown plants.

We confirm that experimental studies on used plants cultivated in the study comply with relevant institutional and national guidelines and legislation effectual at Research Centre for Cultivar Testing^46^.

### 0.1 Pre-sowing stimulation of soy seeds

**Figure 1:**
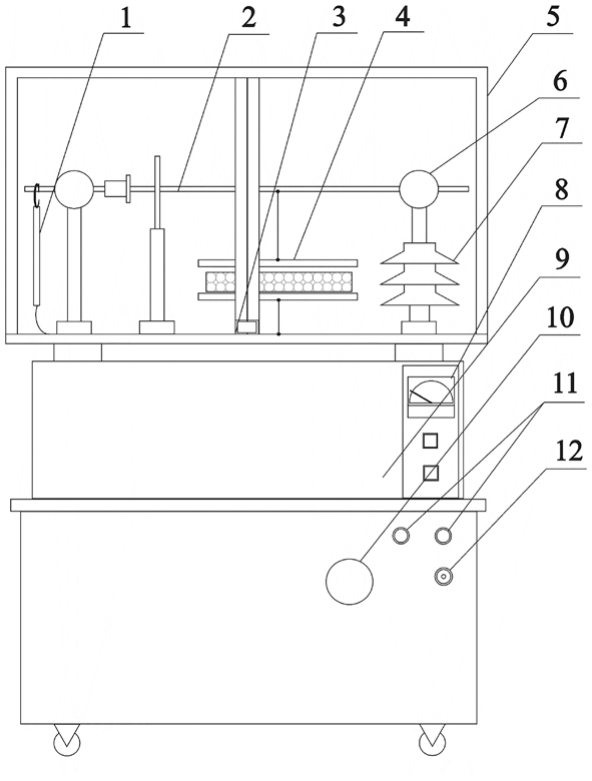
Device used for seed stimulation using an alternating electric field: 1—ground rod, 2—high voltage connection facilitating the switch from constant to alternating current, 3—mechanical safety shut-off, 4—capacitor cladding with the stimulated seeds, 5—high-voltage section cover with doors, 6—high voltage connecting element, 7—high voltage insulator, 8—meter measuring voltage applied to capacitor cladding, 9—Ruhmkorff coil, 10—high voltage control, 11—signal lamps, 12—electrical fuse. Figure by Krzysztof Kornarzyński.

**Figure 2:**
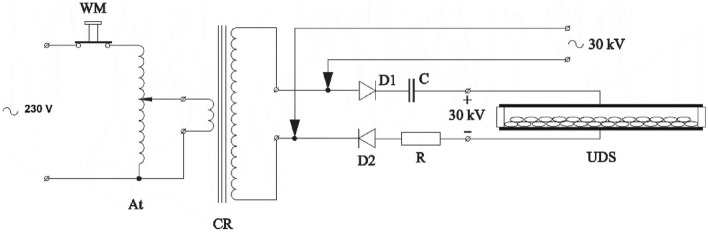
Electrical diagram of the device used for electric field seed stimulation: WM—mechanical safety shut-off, At—autotransformer, CR—Ruhmkorff coil, D1 and D2—rectifying diodes, R—voltage divider, C—capacitor, UDS—seed stimulation system (capacitor). Figure by Krzysztof Kornarzyński.

Before sowing, soy seeds were subjected to stimulation using an alternating magnetic field (magnetic induction B = 30mT for t = 60 s)^47^, constant magnetic field (magnetic induction B = 130mT for t = 17 h)^48^ and alternating electric field (intensity E = 5 kV/cm for t = 60 s)^39,40^(Figs. 1 and 2), respectively designated as: AMF, CMF, and AEF as well as C—control sample (non-stimulated seeds).

### 0.2 Seed germination and plant growth

In the first stage following the ELM stimulation, the soy seeds were sown onto Petri dishes (dia. 15 cm, covered with three layers of filter paper moistened with 15 ml of distilled water). Water was then added throughout the experiment on an ongoing basis. Each Petri dish contained in 4 replications of 30 seeds each (C = 4 replications × 30 seeds = 120 seeds, AMF = 4 replications × 30 seeds = 120 seeds, CMF = 4 replications × 30 seeds = 120 seeds, AEF = 4 replications × 30 seeds = 120 seeds) for each soy cultivar (4 cultivars × 120 seeds = 480 seeds). The experimental samples were as follows: 1—control-unstimulated seeds (C), 2—seeds stimulated with an alternating magnetic field (AMF), 3—seeds stimulated with a constant magnetic field (CMF), 4—seeds stimulated with an alternating electric field (AEF). Moreover, due to the size limitations of the climatic chamber, the experiment was done in two stages: samples of two cultivars were placed in the chamber first, followed by samples of the other two cultivars.

The germination process took place in a climatic chamber under day/night 16*/*8 h illumination, temperatures of 23 °C/12 °C (± 2 °C). The climatic chamber was made of stainless steel and reflected light onto the entire chamber surface. The illumination consisted of 5 warm fluorescent lamps with the total luminous flux of 6,000 lumens. The germination energy (after 5 days) and capacity (after 8 days) were determined in accordance with the applicable ISTA standards^49,50^. The germination energy (*GE*) and capacity (*GC*) are defined as follows:

1 $$ GE(\% ) = \frac{{N_{{5}} }}{N}{1}00\% , $$

2 $$ GC(\% ) = \frac{{N_{{8}} }}{N}{1}00\% , $$

where *N*—number of seeds sown on the dish, *N*_5_—number of seeds germinating after 5 days, *N*_8_—number of seeds germinating after 8 days.

In the second stage of the experiment, seeds were also stimulated with ELM fields and then sown into pots (12 × 11 cm, 1.2 dm^3^). The seeds inside respective pots were spaced by approx. 1.5 × 2.5 cm, the distance from the edge of the pot was 1 cm. The seeds were placed in the soil at the depth of 2 cm. Each pot was filled soil with a pH of 5.5–6.5. The pots were placed on trays filled with water, which ensured its continuous availability. Water in the trays was refilled as needed. The experimental samples were as follows: 1—control-unstimulated seeds (C), 2—seeds stimulated with an alternating magnetic field (AMF), 3—seeds stimulated with a constant magnetic field (CMF), 4—seeds stimulated with an alternating electric field (AEF). Each sample was analysed in 4 replications of 30 seeds each (C = 4 replications × 30 seeds = 120 seeds, AMF = 4 replications × 30 seeds = 120 seeds, CMF = 4 replications × 30 seeds = 120 seeds, AEF = 4 replications × 30 seeds = 120 seeds) for each cultivar (4 cultivars × 120 seeds = 480 seeds). Moreover, due to the size limitations of the climatic chamber, the experiment was done in two stages: samples of two cultivars were placed in the chamber first, followed by samples of the other two cultivars.

In the experiment, parameters including plant emergence (after 5 days), the number of plants (after 8 and 30 days), and green mass (after 30 days) ^49,50^ were determined.

Green mass weighed on a scale with an accuracy of 0*.*01 g.

The plant emergence (*PE*) is defined as

3 $$ PE(\% ) = \frac{{N_{{5}} }}{N}{1}00\% , $$

where *N*—number of seeds sown in pots (units), *N*_5_—number of seeds germinating after 5 days (units).

### Photosynthetic parameters

The greenness index, photosynthetic efficiency (Y (II)), and electron transport rate (ETR) were determined after 15 and 30 days. The measurement was taken at the top of the plan, i.e. its topmost leaves, to account for the best light absorption. The experimental samples were as follows: 1—control-unstimulated seeds (C), 2—seeds stimulated with an alternating magnetic field (AMF), 3—seeds stimulated with a constant magnetic field (CMF), 4—seeds stimulated with an alternating electric field (AEF). Each sample was analysed in 16 replications. The greenness index was measured with the use of a SPAD-502 Chlorophyll Meter^45^. Y (II) and ETR were measured by pulse modulated red light from a light-emitting-diode (LED) with the intensity of 0.84 using a MINI-PAM 2000 WALTZ Photosynthesis Yield Analyser (Germany)^47^. The effective quantum yield (Y (II)) of photochemical energy conversion at the PS II reaction centres was calculated from the following equation:

4 $$ {\text{Y }}\left( {{\text{II}}} \right) \, = \, \left( {{\text{Fm}}^{\prime } \, {-}{\text{ F}}} \right)/{\text{Fm}}^{\prime } , $$

where F—fluorescence yield measured briefly before the onset of the last saturation pulse, Fm’—fluorescence yield reached during the last saturation pulse; normally measured in the presence of actinic light. The relative electron transport rate (ETR) was calculated from the following equation:

5 $$ {\text{ETR }} = {\text{ Y }}\left( {{\text{II}}} \right) \, *{\text{ PAR }}*0.{5 }* \, 0.{84}, $$

where Y (II)—effective quantum yield, PAR—photosynthetically active radiation, the factor of 0.5 accounts for the fact that roughly 50% of all absorbed quanta reach PS II, the standard factor 0.84 corresponds to the fraction of incidental light absorbed by the leaf.

### Kjeldahl’s method

The protein content in leaves was determined with Kjeldahl’s method, following PN-EN_ISO 5983-1:2006/AC:2009^51^. Soy leaves were collected after 30 days. Each sample was weighed to obtain 1g of fresh mass. The method entails mineralisation of the sample, the distillation of ammonia, and titration of the ammonia released. This allows the nitrogen content to be calculated under the protein formula. The experimental samples were as follows: 1—control-unstimulated seeds (C), 2—seeds stimulated with an alternating magnetic field (AMF), 3—seeds stimulated with a constant magnetic field (CMF), 4—seeds stimulated with an alternating electric field (AEF). Each sample was analysed in 3 replications. Leaf samples for protein content measurement were collected from the topmost pars of the plants.

### Statistical analysis

The obtained results were processed by way of ANOVA variance analysis and Fisher test at the significance level of *p* < 0*.*05, using STATISTICA 13.1 software^52^. Significance was determined between the research factors for the respective cultivars and the control (C).

### Machine learning–clustering

We used Python 3.8.5 interpreter and Machine Learning library: Scikit-Learn 0.22.2^53^. Unsupervised learning techniques were used. For detecting clusters, the transformation of data by a manifold learning algorithm—UMAP (Uniform Manifold Approximation and Projection for Dimension Reduction) was used basing on the reference implementation library^54,55^.

### Clustering analysis

Our first attempt to distinguish the impact of electromagnetic fields on soy plants was to use supervised learning for the whole range of data (not averaged). In order to maximise the number of records, only four features were selected: Leaf Greening index SPAD after 15 and 30 days and Y (II) after 15 and 30 days. The data labels were selected as either cultivar, electromagnetic fields, or both. We used multiple classes or a one-class-against-others approach with proper weighting. The standard pipeline was applied that consists of the following steps:

1. Standard scaling of data.
2. Dimensional reduction of data in terms of Principal Component Analysis.
3. Cross-validate models.

with models including^54^ Naive Bayesian, Decision Tree, Support Vector Machine, and Neural Network algorithms of ScikitLearn library^53^. Unfortunately, averaged weighted accuracy^54^ and other metrics in cross-validation^55^ suggest that the effectiveness of prediction is at the level of about 50%.

Due to the ineffectiveness of this approach, we proposed another method that can distinguish various classes based on unsupervised learning (clustering) and nonlinear transformation connected with dimensionality reduction. The redesigned analysis pipeline consists of several steps:

1. Remove outliers.
2. Normalize data.
3. Reduce data dimensionality and nonlinear transform/projection to the three-dimensional space.
4. Compute the optimal number of clusters using the averaged silhouette score.
5. Clustering.

### Designing of clustering algorithm

For the analysis, the algorithms from Scikit Learn 0.22.2.post1 version of Python library^52^ was used.

It was checked that the above data processing pipeline provides the best uniform clusters for the following parameters:

- Greenness index SPAD after 15 and 30 days
- Y (II) after 15 and 30 days
- ETR after 15 and 30 days

The first step is to clean data from the outliers. This step is necessary since, by visual inspection, it was spotted that some of the values look unreliable. The following reasoning explained this: some complicated measurements, such as ETR or Y (II), heavily rely on multiple factors, such as sample preparation and proper measurement procedure that is error-prone. Therefore errors should occur when a high number of measurements is produced. The two standard techniques for outlier/anomaly detection were checked:

- Isolation Forest—sensitive to global outliers
- Local Outliers Factory—isolates local outliers

Both algorithms have their advantages and drawbacks^55^. Isolation Forest was run on specific cultivar types. It was checked that the outliers removed by this approach includes also outliers produced by Local Outliers Factory algorithm, a second common method of outliers detection. Moreover, we noticed that the use of Isolation Forests produces more distinguishable clusters than Local Outliers Factory.

In the next step Hopkins statistics was used to check the clustering tendency in the data. The value is 0*.*8784, which indicates a high tendency to cluster.

Then for each object, standardisation to the mean 0 and STD 1 was made. Since it was checked that the clusters do not separate well upon linear transformation through PCA, it was decided to perform nonlinear transformation with a dimensional reduction to 3 dimensions using UMAP (Uniform Manifold Approximation and Projection for Dimension Reduction)^54,56^. This algorithm has similar or better performance for many cases than the other state-of-the-art nonlinear transformation and dimensionality reduction algorithm t-SNE used commonly for clustering^57^.

Finally, the optimal number of clusters was extracted using two independent techniques: the first one is the maximisation of averaged silhouette score that also provides information on the balance of clusters; the second one is the elbow rule on the SSE vs. the number of clusters plot. After this check, the standard k-Means clustering algorithm was used to identify clusters and check their content.

The result of the procedure is presented in the following section.

## Results and discussion

### Germination and growth of soy plants

The effect of electromagnetic field stimulation on plant germination and growth is shown in Table 1. The results of the statistical analysis illustrate the impact of the main factors: cultivar, electromagnetic fields, and a combination thereof (Table S1.). It was observed that the variables of germination energy and capacity, number of plants and fresh mass after 30 days differed significantly depending on the first factor: the cultivar. In turn, the second factor: electromagnetic fields as well as the combination of the two factors had no significant impact on said variables or plant emergence.

**Table 1:** Seed germination and plant growth.

Cultivars	Electromagnetic fields			
	C	AMF	CMF	AEF
Germination energy (%)				
MAVKA	75.00^a^ ± 5.18	81.67^a^ ± 4.41	65.00^a^ ± 5.69	68.33^a^ ± 6.45
MERLIN	50.00^a^ ± 5.93	51.67^a^ ± 7.39	60.00^a^ ± 6.38	52.50^a^ ± 1.60
VIOLETTA	54.17^a^ ± 3.4	56.67^a^ ± 3.60	60.00^a^ ± 3.04	40.83^a^ ± 10.83
ANUSZKA	37.50^a^ ± 5.51	44.17^a^ ± 2.50	44.17^a^ ± 4.98	36.67^a^ ± 5.77
Germination capacity (%)				
MAVKA	75.00^a^ ± 5.18	82.50^a^ ± 3.70	65.00^a^ ± 5.69	68.33^a^ ± 6.45
MERLIN	50.00^a^ ± 5.93	51.67^a^ ± 7.39	60.00^a^ ± 6.38	52.50^a^ ± 1.60
VIOLETTA	61.67^a^ ± 2.15	58.33^a^ ± 4.41	57.50^a^ ± 3.15	45.00^b^ ± 10.05
ANUSZKA	44.17^a^ ± 4.97	45.00^a^ ± 2.15	37.50^a^ ± 6.58	37.50^a^ ± 5.99
Plants emergence (%)				
MAVKA	16.67^a^ ± 2.72	33.33^a^ ± 12.84	20.00^a^ ± 6.80	16.67^a^ ± 5.61
MERLIN	30.83^a^ ± 5.67	21.67^a^ ± 6.45	16.67^a^ ± 6.94	30.83^a^ ± 7.12
VIOLETTA	12.50^a^ ± 5.51	21.67^a^ ± 6.87	15.83^a^ ± 1.60	16.67^a^ ± 3.04
ANUSZKA	18.33^a^ ± 5.18	23.33^a^ ± 5.61	15.83^a^ ± 3.70	10.00^a^ ± 3.60
Number of plants after 30 days				
MAVKA	7.50^a^ ± 1.85	11.75^a^ ± 4.31	8.25^a^ ± 1.93	6.00^a^ ± 2.12
MERLIN	11.25^a^ ± 2.56	8.00^a^ ± 1.78	6.75^b^ ± 2.81	12.50^a^ ± 2.06
VIOLETTA	2.00^a^ ± 1.08	5.75^a^ ± 2.14	6.75^b^ ± 2.81	12.50^a^ ± 2.06
ANUSZKA	5.50^a^ ± 1.55	6.00^a^ ± 1.58	5.00^a^ ± 1.35	4.00^a^ ± 1.87
Fresh mass of seedlings (g pot^−1^)				
MAVKA	8.83^a^ ± 2.64	13.08^a^ ± 4.98	15.12^a^ ± 3.37	7.11^a^ ± 2.44
MERLIN	19.44^a^ ± 2.08	13.29^a^ ± 1.71	9.30^b^ ± 4.11	12.69^a^ ± 2.20
VIOLETTA	2.30^a^ ± 1.29	5.36^a^ ± 2.22	2.73^a^ ± 0.47	4.43^a^ ± 1.06
ANUSZKA	5.00^a^ ± 1.39	6.29^a^ ± 2.19	4.50^a^ ± 1.19	3.21^a^ ± 1.48

C—control (unstimulated seeds), AMF—alternating magnetic field, CMF—constant magnetic field, AEF—alternating electric field ± standard deviation, a–b—different letters in respective rows indicate statistical differences between tested samples and the control (p < 0*.*05), n = 4.

Under the influence of the ELM fields, there was a noticeable increase in germination energy and capacity for the MERLIN cultivar, as compared to the control (C). However, the observed differences were not statistically significant (p > 0.05). Moreover, stimulation with the alternating electric field caused a significant, 27% decrease in the germination capacity of VIOLETTA plants, as compared to the control (C). The germination energy increased slightly by 3% (MERLIN), 5% (VIOLETTA, 9% (MAVKA), and 18% (ANUSZKA) under the influence of the alternating magnetic field (AMF) relative to the control (C). Simultaneously, the germination capacity increased slightly after the AMF treatment, respectively by: 2% (ANUSZKA), 3% (MERLIN), and 10% (MAVKA), as compared to the reference samples (C). The best effects were observed for constant magnetic field stimulation and ANUSZKA and MERLIN cultivars for which the germination energy increased by between 18 and 20%. No statistically significant differences were observed between samples stimulated with the constant magnetic field and the control (unstimulated seeds), as the p value was > 0.05. Stimulation with alternating magnetic field (AMF) caused an insignificant increase in emergence and number of plants after 30 days, as well as fresh mass of seedlings, for all plants except the MERLIN cultivar. The effect of ELM field stimulation increased the emergence in the case of the VIOLETTA cultivar, however, without significant statistical differenced (p > 0.05). After 30 days, the number of plants and fresh weight increased for alternating and constant magnetic field for two cultivars (MAVKA, VIOLETTA). Nonetheless, no statistically significant differences were recorded (p > 0.05). Iqbal et al.^58^ reported that the use of a magnetic field with the magnetic induction of 60 and 180 mT significantly improved the germination parameters in pea. Vashisth and Nagrajan^59^ used constant magnetic fields of between 0 and 250mT for 1–4 h, which improved the germination and fresh mass of chickpea. Similar effects were reported for corn by Bilalis et al. ^60^ who stimulated seeds with a pulsating magnetic field for 0, 15, 30, and 45 min.

In literature reports, germination and fresh mass of shoots increased under the influence of soy seed stimulation with an alternating magnetic field (AMF) (*B* = 1500nT, frequency 10 and 100 Hz)^61^. The quality of soy seeds was also improved by regulating the metabolism of storage proteins and fatty acids using AMF (*B* = 1500nT, 10 Hz)^36^. In the studies of Shine et al.^39,62^ an improvement in the germination of soy seeds, an increase in the production of reactive oxygen species (ROS), and peroxidase activity in the biochemical basis of better seedling germination and growth under the influence of permanent magnetic fields (magnetic induction 150 and 200mT for 60 min) were noted. On the other hand, Garciá et al.^63^ obtained an increased germination capacity of soy seeds treated with a constant CMF field (100 and 150 mT). Atak et al.^19^ conducted a stimulation of soy seeds in a system of 10 permanent magnets, under the influence of which shoots and roots grew. Aladjadjiyan^64^ reported an increase in lentil germination under the influence of 150 mT constant magnetic field. Our studies also revealed improvement in soy germination by up to 20% (MERLIN).

Costanzo^65^ studies on the effect of low-frequency electric fields on soy seedlings indicate that electric fields of 3600 and 1800 V/m increase the length of seedlings by approximately 12% and 8%, respectively. Bean seeds infected with a fungus with a germination capacity of 30%, subjected to an electric field (50 Hz, *E* = 2−6 kV/cm, *t* = 1−30 s), after which 99% more seeds sprouted^66^. On the other hand, studies on the cultivation of wheat under the influence of an alternating electric field (AEF) (*E* = 6 kVm^−1^) caused a high chromosomal abnormality of flower buds and an increase in the number of dead grains, which contributes to a decrease in weight and number of grains per spike^67^. Field stimulation high-intensity electricity (*E* = 5 and 10 kV/cm, 50 Hz) reduces the germination and seed yield of tomato^68^ and radish^34^. Literature also provides evidence for increased germination of carrot (24 %), reddish, beat (12%), and barley (9%) after electric field seed stimulation^69^. The use of electric fields of varying intensity (0 kV/m, 2 kV/m, 10 kV/m) led to an increase in germination speed correlated with growing field intensity^70^. In our studies, the germination energy of soy increased by 5% (MERLIN) under the influence of electric field stimulation.

### Soy leaf photosynthetic parameters

Table 2 shows the effect of electromagnetic field stimulation on the photosynthetic parameters of soybean leaves. The data presented in Table S2. clearly indicate that the factors differentiating the variables of photosynthetic efficiency after 30 days as well as greenness index after 15 and 30 days included the soy cultivar, electromagnetic stimulation, and the interaction between the same. Simultaneously, no significant influence of the differentiating factors was observed in terms of the other photosynthetic parameters, (Y (II) and ETR after 15 days).

**Table 2:** Photosynthetic parameters of soy leaves.

Cultivars	Electromagnetic fields			
	C	AMF	CMF	AEF
Photosynthetic efficiency (Y II) after 15 days				
MAVKA	0.786^b^ ± 0.008	0.797^a^ ± 0.007	0.789^b^ ± 0.006	0.798^a^ ± 0.005
MERLIN	0.790^b^ ± 0.004	0.802^a^ ± 0.005	0.780^b^ ± 0.005	0.800^a^ ± 0.006
VIOLETTA	0.788^a^ ± 0.013	0.793^a^ ± 0.011	0.788^a^ ± 0.009	0.785^a^ ± 0.007
ANUSZKA	0.678^b^ ± 0.053	0.772^a^ ± 0.028	0.752^a^ ± 0.057	0.793^a^ ± 0.010
Photosynthetic efficiency (Y II) after 30 days				
MAVKA	0.666^b^ ± 0.048	0.778^a^ ± 0.031	0.780^a^ ± 0.011	0.793^a^ ± 0.010
MERLIN	0.787^a^ ± 0.012	0.792^a^ ± 0.009	0.789^a^ ± 0.008	0.784^a^ ± 0.007
VIOLETTA	0.775^a^ ± 0.011	0.780^a^ ± 0.006	0,761^a^ ± 0.030	0.771^a^ ± 0.011
ANUSZKA	0.733^a^ ± 0.069	0.743^a^ ± 0.068	0.765^a^ ± 0.008	0.757^a^ ± 0.20
Electron transport rate (ETR) after 15 days				
MAVKA	0.66^a^ ± 0.05	0.70^a^ ± 0.00	0.66^a^ ± 0.13	0.70^a^ ± 0.00
MERLIN	0.30^a^ ± 0.93	0.50^a^ ± 0.21	0.30^b^ ± 0.00	0.50^a^ ± 0.21
VIOLETTA	3.13^b^ ± 0.93	2.90^a^ ± 0.59	2.92^a^ ± 0.63	1.66^a^ ± 0.24
ANUSZKA	2.52^a^ ± 0.33	3.04^a^ ± 0.47	2.41^a^ ± 1.19	3.15^a^ ± 0.69
Electron transport rate (ETR) after 30 days				
MAVKA	2.58^a^ ± 0.39	2.99^a^ ± 0.39	3.12^a^ ± 0.70	3.05^a^ ± 0.82
MERLIN	3.23^a^ ± 0.80	2.73^a^ ± 0.67	2.79^a^ ± 0.68	1.72^b^ ± 0.37
VIOLETTA	2.89^a^ ± 0.51	2.20^b^ ± 0.67	2.21^b^ ± 0.61	1.94^b^ ± 0.22
ANUSZKA	2.15^a^ ± 0.67	2.27^a^ ± 0.68	2.25^a^ ± 0.52	2.39^a^ ± 0.45
Greenness index (SPAD) after 15 days				
MAVKA	31^b^ ± 5	27^b^ ± 5	40^a^ ± 4	32^b^ ± 5
MERLIN	38^a^ ± 6	36^b^ ± 4	42^a^ ± 4	33^b^ ± 6
VIOLETTA	27^a^ ± 3	26^a^ ± 2	26^a^ ± 3	25^a^ ± 3
ANUSZKA	28^a^ ± 2	29^a^ ± 3	28^a^ ± 3	26^a^ ± 2
Greenness index (SPAD) after 30 days				
MAVKA	26^b^ ± 3	29^a^ ± 1	41^a^ ± 4	39^a^ ± 4
MERLIN	39^a^ ± 5	36^b^ ± 4	42^a^ ± 4	33^b^ ± 6
VIOLETTA	29^a^ ± 5	29^a^ ± 3	27^b^ ± 3	28^a^ ± 3
ANUSZKA	31^a^ ± 4	31^a^ ± 3	31^a^ ± 2	30a ± 3

C—control (unstimulated seeds), AMF—alternating magnetic field, CMF—constant magnetic field, AEF—alternating electric field ± standard deviation, a–b—different letters in respective rows indicate statistical differences between tested samples and the control (p < 0*.*05), n = 16.

Stimulation with fields, especially alternating magnetic field (AMF), increased photosynthesis efficiency (Y (II)) after 15 and 30 days, regardless of the soybean variety. The applied electromagnetic field in the case of the MAVKA variety significantly increased the efficiency of photosynthesis after 30 days. The highest increases in Y (II) photosynthesis of 17% (AMF and CMF), 19% (AEF), and electron transport (ETR) of 16% (AMF), 18% (AEF) and 21% (CMF) were recorded after 30 days compared to the control group C. In turn, the electron transport rate (for MAVKA, ETR) after 30 days returned no statistically significant differences (p > 0.05). A significant decrease in terms of the ETR after 30 days was observed for the Violetta cultivar under the influence of ELM stimulation, as compared to the reference samples (C). The greenness index (SPAD) after 15 and 30 days increased under the influence of constant magnetic field stimulation for varieties MAVKA, and alternating magnetic field after 30 days for variety MAVKA. The highest values of the SPAD index after 30 days were recorded for the MAVKA variety in all the fields used, respectively: 12% (AMF), 50% (AEF), and 58% (CMF) relative to the control. We also observed an increase in the greenness index in the case of the MERLIN cultivar (CMF), but the discrepancies were not statistically significant as the p value was > 0.05.

In another study pertaining to alternating and constant magnetic fields, the content of chlorophyll in soy seedlings was higher by, respectively, 21% and 38% compared to the control.^7^

Under correct growth conditions, plants typically reach the Y (II) photosynthetic activity level of approx. 0*.*856^42^, whereas in our study, the photochemical activity of the analysed soya plants was within the range of 0*.*666–0*.*802. In the studies of Shine et al.^37^, it was found that stimulation with a constant magnetic field had a positive effect on the efficiency of photosynthesis. The electron transport flow was achieved up to 0*.*525, and in our studies, it was up to 3*.*23 (ETR). Whereas Michalak et al.^71^ found an increase in SPAD chlorophyll content in MERLIN soy seedlings to 27% (CMF, 12 min. exposure time) and 21% (AMF, 2*.*5 min. exposure time), and its decrease by 9*.*8% (CMF, 6 min. exposure time) and 2*.*2% (AMF, 5 min. exposure time). A similar result was obtained in our study for the alternating magnetic field, where the pigment significantly decreased by approximately 5% and 8% in plants of the MERLIN cultivar (after 15 and 30 days). In our plants, after 30 days in MERLIN cultivar, we observed an insignificant increase in chlorophyll concentration by 7.7% for CMF (17 h), an almost identical effect was obtained by Michalak et al.^71^ 5*.*7% (CMF, 5 min.).

Commoner et al.^72^ attributed the decrease in the content of pigments to their chemical composition with unpaired electrons possessing a magnetic moment, which plays a key role in the electron transfer occurring during chemical reactions. Electrons with magnetic moments may be oriented in the outer field whose energy is absorbed due to interactions between the field and the magnetic moment of the unpaired electrons. Since chloroplasts have their own magnetic moments, it is possible that under the influence of large doses of absorbed energy, a distortion of photosynthetic pigment synthesis may occur^73,74^.

Stimulation with electric fields with the intensity of 2 kV/m and 10 kV/m significantly increased the content of chlorophyll in soy^70^ compared to control group plants. A similar effect was also observed in our study where the greenness index significantly increased in 30-day MAVKA plants (50%). In studies conducted by other authors, increased content of chlorophyll was reported in bean plants under the influence of an electric field with the intensity of 6 kV/cm^75^.

Stimulation with electric fields with the intensity of 2 kV/m and 10 kV/m significantly increased the content of chlorophyll in soy^70^ compared to control group plants. A similar effect was also observed in our study where the greenness index significantly increased in 30-day MAVKA plants (50%). In studies conducted by other authors, increased content of chlorophyll was reported in bean plants under the influence of an electric field with the intensity of 6 kV/cm^75^.

### Protein content in soy leaves

Table 3 presents the mean content of protein in the leaves under the influence of ELM stimulation. The data presented in Table S3 clearly indicate that the content of protein in soy leaves differed significantly depending on the soy cultivar, electromagnetic filed stimulation, and interaction between the same. Stimulation using ELM fields had a noticeable, albeit statistically insignificant, impact in terms of increased protein content measured in the ANUSZKA cultivar (AMF, AEF) as well as decreased content of the same in VIOLETTA plants, as compared to the control. At the same time, treatment with the constant magnetic field (CMF) significantly increased the protein content in the leaves of ANUSZKA, MERLIN, and MAVKA plants. The respective recorded rise was by: 5%, 13%, and 16% relative to the control. The alternating magnetic field (AMF) caused a statistically insignificant increase in the protein content in ANUSZKA and MERLIN plants, approximately by 3–4% as compared to the control (C). The protein content in the leaves of most cultivars (MAVKA, MERLIN, ANUSZKA) remained at levels similar to the control (C) under the influence of the alternating electric field.

**Table 3:** The protein content of soybean leaves.

Cultivars	Electromagnetic fields			
	C	AMF	CMF	AEF
Protein content (%)				
MAVKA	26^b^ ± 1	23^b^ ± 2	42^a^ ± 4	25^b^ ± 1
MERLIN	26^b^ ± 2	30^b^ ± 3	39^a^ ± 3	26^b^ ± 1
VIOLETTA	27^a^ ± 1	25^a^ ± 1	26^a^ ± 1	26^a^ ± 2
ANUSZKA	27^b^ ± 2	30^b^ ± 2	32^a^ ± 1	30^b^ ± 2

C—control (unstimulated seeds), AMF—alternating magnetic field, CMF—constant magnetic field, AEF—alternating electric field ± standard deviation, a–b—different letters in respective rows indicate statistical differences between tested samples and the control (p < 0*.*05), n = 3.

Mroczek-Zdyrska et al.^48^ observed a similar increase (13–22%) in the content of protein in the sprouts and roots of lupin grown from seeds stimulated with a 130 mT magnetic field. Furthermore, Asghar et al. ^37^ observed that the concentration of protein and chlorophyll increased in the seeds and seedlings of soy after magnetic field and laser light stimulation. The least amounts of protein were observed in the AMF sample (2–3%) (VIOLETTA, MAVKA), however without statistically significant differences (statistical p value > 0.05). Research results by Radhakrishnan^34^ showed that after stimulation of soy seeds with AMF (1500nT and 10 Hz), an increase in protein concentration in plants was observed. Similar statistically insignificant effects in terms of increased protein content in leaves in two cultivars: MERLIN and ANUSZKA, induced by alternating the magnetic field, were obtained in our research. In turn, Kumar et al.^74^, after stimulating soy seeds stored for 6 months with CMF, observed elevated protein concentration in the yield as compared to the control, which is consistent with our results obtained for the MAVKA, MERLIN, and ANUSZKA cultivars where the CMF was found to have the highest positive influence relative to the control. Magnetic fields with low induction values facilitate intensified binding or breakup of proteins in plant roots to a greater extent than in seedlings^28^. Based on the results recorded in our study, it can be concluded that the decrease in protein content observed in some cultivars may have been related to the accumulation of large quantities thereof in the roots of the plants. In another study, soya seeds were subjected to the effects of an alternating magnetic field (AMF with 1*.*5 µT induction, 1*.*0 Hz)^5^, where the explants of soya germinal axis were implanted into a saline medium containing 10 nM NaCl. It was observed that the combined effects of AMF and salt significantly increases the fresh weight of the callus and protein. This suggests that magnetic field stimulation is an effective and environmentally friendly method of improving seed quality. In our study, the content of protein increased under the influence of the electric field stimulation for the ANUSZKA cultivar, while in the other plants it remained similar to that in the control sample. Li et al. observed a significantly higher protein content in soy treated with 2 kV/m electric field as compared to 10 kV/m field and relative to the control (0 kV/m)^70^.

Stimulation with electromagnetic fields can either increase or decrease the rate of plant germination and growth as well as levels of photosynthetic parameters^35–39,47^. If too much energy is absorbed by chloroplasts, the plant’s development is hindered. Chloroplasts are characterised by their own magnetic moment which can be distorted due excessive energy during synthesis of photosynthetic pigments^76^. 5It is generally accepted that the content of chlorophyll is a key factor in plant development^77^. It is a marker of leaf ageing as well as a potential indicator of environmental stress. Moreover, chlorophyll is involved in the process of photosynthesis taking place in respective stages of a plant’s life, allowing it to adapt to varying, sometimes quite harsh environmental conditions. Photosynthesis takes place within chloroplasts in two stages of light-dependent and light-independent reactions. Light energy is converted and stored in the form of chemical energy necessary for the subsequent light-independent reactions^78^. However, excessive light absorption cannot cause damage to the photosynthetic apparatus^79^. Plants utilise a variety of mechanisms that allow them to cope with radiative stress, e.g.: barley plants grown under different light intensity conditions have been observed to show differences in terms of the PSII fluorescence index^80^. Other mechanisms allowing plants to protect their photosynthetic apparatuses include slowing down electron transport and partially degrading the key protein D1^81^.

In our study, the content of protein statistically insignificantly increased under the influence of the electric field stimulation for the ANUSZKA cultivar, while in the other plants it remained similar to that in the control sample.

Literature reports indicate that magnetic fields can influence seed germination by stimulating the activity of proteins and enzymes in biochemical processes taking place in seeds. Moreover, they facilitate faster growth of roots and sprouts as well as increased content of photosynthetic pigments and nutrients^82^. Plants utilise blue light receptors, so-called cryptochromes that create a pair of radicals when excited. This allows proteins to act as radical-based magnetic sensors. Cryptochromes present in plants control their development and growth^83^. However, the exact mechanism through which magnetic fields affect said processes remains unclear. Some researchers associate germination variability with biochemical changes or impact on enzyme activity. Aksenov et al.^84^ observed that wheat seeds were faster to swell and release enzymes under the influence of low-induction magnetic fields, which induced seed dormancy. At later stages, the effects of magnetic field stimulation were considerably less apparent. Another hypothesis suggests that magnetic fields interact with ionic currents inside membrane cells, which leads to changes in ionic concentrations and osmotic pressure, and consequently water intake by the seed. The effects of magnetic field stimulation are observed primarily in terms of the speed of seed germination, which in turn influences the overall plant growth and resulting yields^85^.

Despite numerous the studies conducted to date, the exact mechanism of magnetic field influence remains unknown. It is noteworthy in this context that soy belongs to the Fabaceae family. The seeds of such plants struggle with poor or hindered germination due to the character of their dormancy period which is not interrupted even in optimum environmental conditions. Among other possible reasons, the absolute dormancy is due to the hardness and water-impermeability of the seed coat. Still, the exact mechanism responsible is yet to be determined. Despite the ongoing scientific and technological advances, many secrets of the mechanisms related to plant growth remain undiscovered. For this reason, we were driven to explore this research problem, which our group has been analysing for over a dozen years.

### Clustering analysis of cultivars

#### MAVKA

This class contains 51 records. After scaling, UMAP transformation, and projection, the averaged silhouette score was optimised for 15 clusters. The k-Means clustering is presented in Figure S1 in Supplementary Materials. and the clusters are presented in Table 4. It is visible that most of the clusters are clean, and therefore our approach can distinguish seeds exposed to different electromagnetic fields.

**Table 4:** Content of the clusters for MAVKA cultivar.

Cluster number	Physical factor
0	AEF, AEF, AEF, AEF
1	AEF, AEF, AEF, C, C, CMF, CMF, CMF
2	AMF, AMF
3	AEF, AEF, CMF
4	AEF, CMF
5	AEF, CMF, CMF, CMF, CMF
6	CMF, CMF
7	AMF, AMF, AMF, AMF
8	AEF, C, C, C, C, C
9	AMF, AMF, C
10	C, C, C
11	CMF, CMF
12	AMF, AMF
13	AEF, AMF, C
14	AMF, C

#### MERLIN

This class contains 47 samples. A similar procedure, as above, provided 13 clusters with the content in Table 5, and k-Means clustering is presented in Figure S2 in Supplementary Materials. The obtained clusters are also distinguished.

**Table 5:** Content of the clusters for MERLIN cultivar.

Cluster number	Physical factor
0	AEF, AEF, AEF, AMF
1	AEF, AEF, AEF, AEF
2	C, C, C, C, C, C
3	AEF, C, CMF, CMF, CMF, CMF, CMF, CMF, CMF
4	AMF, AMF, AMF
5	AEF, AEF, AEF
6	C, C
7	AEF, AMF, AMF, AMF
8	C, C
9	AMF, AMF, AMF
10	AMF, C, C
11	CMF, CMF
12	AMF, AMF

#### VIOLETTA

The sample contains 48 records. The cluster content is presented in Table 6, and k-Means clustering is presented in Figure S3 in Supplementary Materials. In this case, some clusters are mixed with no distinguished leading factor, e.g., clusters no. 7 and 9.

**Table 6:** Content of the clusters for VIOLETTA cultivar.

Cluster number	Physical factor
0	AEF, AEF, AEF, AEF, AEF, AEF, AEF, CMF, CMF, CMF
1	AMF, AMF, AMF, AMF, C, C, CMF, CMF
2	C, C, C, C, C, CMF
3	AMF, AMF, AMF, AMF, CMF
4	AEF, AEF, AEF, C, CMF
5	AEF, C, C, CMF
6	CMF, CMF, CMF
7	AMF, C, CMF
8	AMF, AMF
9	AEF, CMF

#### ANUSZKA

For this cultivar, there are 46 records, and the optimal number of clusters is determined to be 10. The content of clusters is presented in Table 7, and k-Means clustering is presented in Figure S4 in Supplementary Materials. In this case, the clusters are the most nonuniform. The proposed method can distinguish some clear clusters, but most are nonuniform.

**Table 7:** Content of the clusters for ANUSZKA cultivar.

Cluster number	Physical factor
0	AEF, AEF, AMF
1	AEF, CMF
2	AMF, AMF, AMF
3	AEF, AEF, AEF, AMF, C, C, CMF, CMF
4	AMF, AMF, C, CMF
5	AMF, AMF, C, C, C, C, CMF, CMF, CMF, CMF
6	AMF, AMF, AMF
7	AEF, AEF, AEF, AEF, AMF, C, C, CMF
8	AMF, C, CMF
9	C, C

The method to distinguish plants under various electromagnetic fields was proposed and checked on specific examples. The first two cultivars (MAVKA, MERLIN) provide relatively high predictive power, indicating that the electromagnetic field alters Yield II, ETR, and leaf greenness parameters. This indicates a potential field to explore in more detailed experiments with higher statistics. VIOLETTA shows a less visible distinction under ELM fields. The distinction is smallest for ANUSZKA cultivar. This can suggest that ANUSZKA is less susceptible to the used fields.

The proposed processing/clustering pipeline can be used in more advanced studies of the influence of external factors on plants growth. Moreover, the failure of standard supervised ML methods indicates that the influence of ELM fields can be masked by some nonlinear relations between plats parameters, that more advanced transformation algorithms, like UMAP, can unmask.

## Conclusions

Relative to the control, the Violetta cultivar showed an increase in plant emergence, number of plants after 30 days, and fresh mass, in which it stood out from the other cultivars analysed. This was a very interesting observation, and the topic should definitely be explored further. In the future, we hope to be able to conduct a larger scale, field experiment in this context. Moreover, increased photosynthetic efficiency was observed after 15 and 30 days in all analysed cultivars after alternating magnetic field treatment. The values of plant emergence, number of plants, and fresh mass after 30 days were also increased under the influence of AMF stimulation in all plants except the MERLIN cultivar. Furthermore, one should also point out the significant influence of constant magnetic fields on the photosynthetic efficiency, greenness index, and protein content in the leaves of 30-day MAVKA plants, which increased by, respectively: 17%, 58%, and 62%. It is noteworthy that the cultivar showed an increase of 10% in terms of the number of plants, 20% in terms of plant emergence, and 71% in terms of fresh mass.

It can be concluded that changes in the parameters of germination and plant growth, as well as the chlorophyll and protein content, depended both on the particular ELM treatment employed and the soy cultivar in question. It is noteworthy that physical factors may indeed be used to stimulate soy seeds in a non-invasive and environmentally friendly manner as they introduce no changes to the natural agricultural ecosystem. Stimulation can improve germination, fresh mass, and photosynthetic efficiency, but further research in this context is definitely needed. Moreover, we proposed a clustering algorithm that can be used to examine the nonlinear relation between external stimuli.

### Supplementary Information

Supplementary Information.

## Data Availability

Data will be available on request. Correspondence and requests for materials should be addressed to A.D.-H.
