# Supplementary material for: Stimulation of soy seeds using environmentally friendly magnetic and electric fields
Source: Sci Rep. 2023 Oct 23;13:18085. doi: 10.1038/s41598-023-45134-y (PMC10593769; doi:10.1038/s41598-023-45134-y)
Supplement: Supplementary file 1 — Supplementary Information. [file 41598_2023_45134_MOESM1_ESM.pdf]

Variable and source of variation	df	F	p
Germination energy			
Cultivar (A)	3	22.18	0.000
ELM field (B)	3	2.04	0.121
Cultivar x ELM field (A x B)	9	1.05	0.419
Germination capacity			
Cultivar (A)	3	22.10	0.000
ELM field (B)	3	1.81	0.158
Cultivar x ELM field (A x B)	9	1.02	0.436
Plant emergence			
Cultivar (A)	3	1.738	0.172
ELM field (B)	3	1.275	0.292
Cultivar x ELM field (A x B)	9	0.981	0.467
Number of plants after 30 days			
Cultivar (A)	3	6.658	0.001
ELM field (B)	3	0.713	0.549
Cultivar x ELM field (A x B)	9	1.051	0.415
Fresh mass of seedlings			
Cultivar (A)	3	15.01	0.000
ELM field (B)	3	0.97	0.416
Cultivar x ELM field (A x B)	9	1.37	0.229
df – degrees of freedom, F –Fischer test, p – significance level $p < 0.05$			

Table S1. Two-way ANOVA illustrating the impact of two primary factors: cultivars, electromagnetic fields, and interactions between the same on the germination energy and capacity, plant emergence and number of plants after 30 days, as well as the fresh mass of seedlings.

Variable and source of variation	df	F	p
Photosynthetic efficiency (Y II) after 15 days			
Cultivar (A)	3	1.629	0.183
ELM field (B)	3	0.262	0.853
Cultivar x ELM field (A x B)	9	2.106	0.030
Photosynthetic efficiency (Y II) after 30 days			
Cultivar (A)	3	12.49	0.000
ELM field (B)	3	8.45	0.000
Cultivar x ELM field (A x B)	9	3.26	0.001
Electron transport rate (ETR) after 15 days			
Cultivar (A)	3	1.483	0.220
ELM field (B)	3	0.544	0.653
Cultivar x ELM field (A x B)	9	1.335	0.219
Electron transport rate (ETR) after 30 days			
Cultivar (A)	3	3.584	0.014
ELM field (B)	3	2.528	0.058
Cultivar x ELM field (A x B)	9	1.894	0.053
Greenness index (SPAD) after 15 days			
Cultivar (A)	3	84.01	0.000
ELM field (B)	3	11.56	0.000
Cultivar x ELM field (A x B)	9	8.66	0.000
Greenness index (SPAD) after 30 days			
Cultivar (A)	3	72.57	0.000
ELM field (B)	3	14.07	0.000
Cultivar x ELM field (A x B)	9	23.32	0.000
df – degrees of freedom, F –Fischer test, p – significance level $p < 0.05$			

Tabela S2. Two-way ANOVA illustrating the impact of two primary factors: cultivars, electromagnetic fields, and interactions between the same on Photosynthetic efficiency (Y II) after 15 and 30 days, Electron transport rate (ETR) after 15 and 30 days, Greenness index (SPAD) after 15 and 30 days.

Variable and source of variation	df	F	p
Protein content			
Cultivar (A)	3	11.62	0.000
ELM field (B)	3	52.11	0.000
Cultivar x ELM field (A x B)	9	14.84	0.000
df – degrees of freedom, F –Fischer test, p – significance level p < 0.05			

Tabela S3. Two-way ANOVA illustrating the impact of two primary factors: cultivars, electromagnetic fields, and interactions between the same on the content of protein.

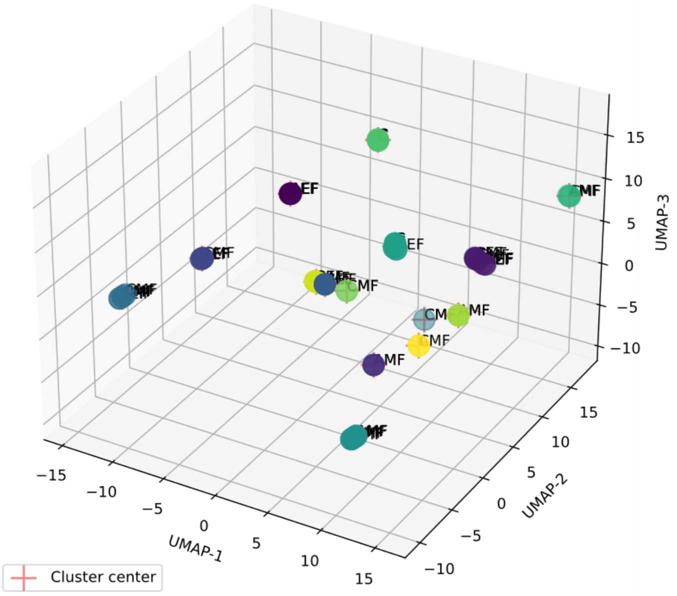

Figure S1. 15 clusters for MAVKA

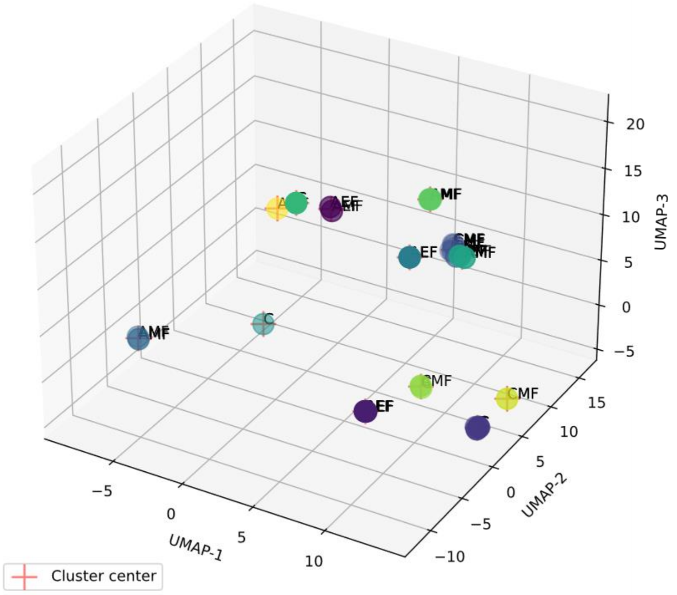

Figure S2. 13 clusters for MERLIN

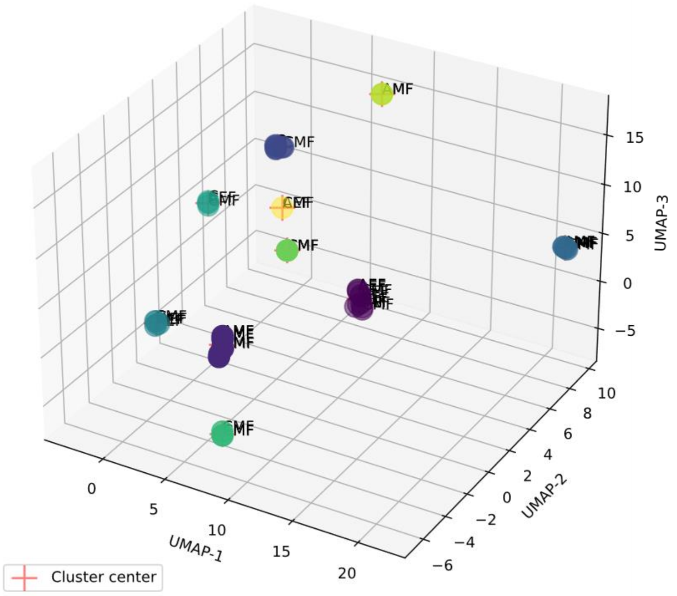

Figure S3. 10 clusters for VIOLETTA

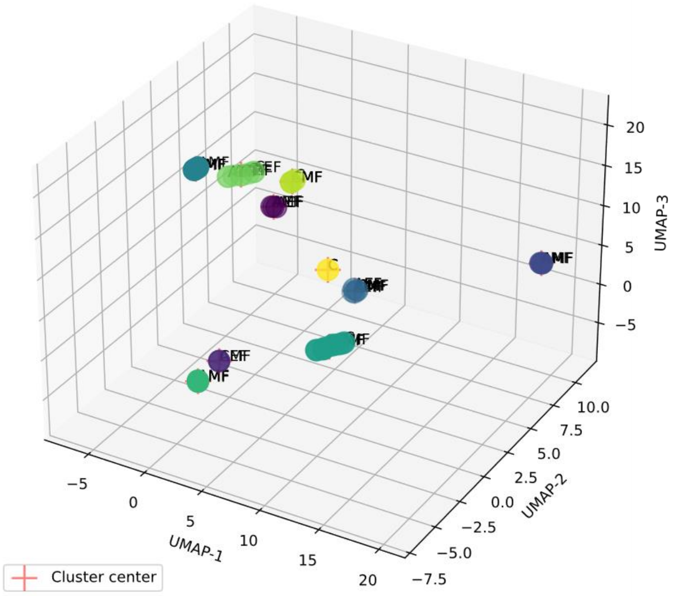

Figure S4. 10 clusters for ANUSZKA
